# Selective Serotonin Reuptake Inhibitors (SSRIs) and Surgical Bleeding in Plastic Surgery: A Systematic Review

**DOI:** 10.7759/cureus.79639

**Published:** 2025-02-25

**Authors:** Nathan John, Francisco A Ferri, Emanuella M Brito, Maya N Devineni, Martin I Newman

**Affiliations:** 1 General Surgery, Cleveland Clinic Florida, Weston, USA; 2 Plastic Surgery, Cleveland Clinic Florida, Weston, USA; 3 Medicine, Nova Southeastern University Dr. Kiran C. Patel College of Osteopathic Medicine, Fort Lauderdale, USA; 4 Medicine, Midwestern University Arizona College of Osteopathic Medicine, Glendale, USA; 5 Plastic and Reconstructive Surgery, Cleveland Clinic Florida, Weston, USA

**Keywords:** antidepressant therapy, bleeding, bleeding risk, plastic and reconstructive surgery, plastic surgery, post-operative bleeding, selective serotonin reuptake inhibitor (ssri), ssri side effects

## Abstract

Selective serotonin reuptake inhibitors (SSRIs) are first-line pharmacotherapy for various psychiatric disorders. These medications adversely affect hemostasis by limiting serotonin reuptake crucial for platelet aggregation. Increased bleeding risk vs SSRI discontinuation syndrome may have important clinical considerations for plastic and reconstructive surgeries (PRSs). With the increasing use of SSRIs, understanding the associated risk of bleeding in soft-tissue surgeries is crucial for optimizing patient outcomes and ensuring safety. In this systematic review, the National Center for Biotechnology Information (NCBI) and Embase databases were searched for publications addressing SSRI consumption and bleeding outcomes in PRS and soft-tissue procedures. Five retrospective cohort studies on SSRIs and bleeding were identified, differing in surgical types, study designs, and reporting of bleeding outcomes, precluding meta-analysis. Post-operative bleeding rates ranged from 1.9% to 2.6%. A U.S. study on breast cosmetic procedures reported a 4.14-fold increase in hematoma reoperations (odds ratio (OR) 4.14; 95% CI 1.90-9.04), while a Danish study on breast oncologic procedures showed a 2.7-fold increased risk (OR 2.7; 95% CI 1.6-4.4). Another Danish study found no increased risk of post-operative blood transfusion (OR 1.2; 95% CI 0.7-1.9). Two U.S. studies on facial procedures found no significant differences in bleeding events, though both had low statistical power. Overall, the risk of significant bleeding complications in breast and facial PRS procedures appears low, with SSRI use increasing hematoma reoperation risk for breast procedures to less than 5%, without evidence of life-threatening bleeding. Conclusions regarding SSRI-related bleeding in facial plastic surgery are limited due to the overall quality of existing studies, which often rely on case reports rather than higher-quality research designs, such as cohort or prospective analyses. Therefore, further high-level evaluations of SSRI-related bleeding are necessary, particularly focusing on procedures involving the face, extremities, and flap surgeries.

## Introduction and background

Major depressive disorder (MDD; “depression”) is a mood disorder presenting with a variety of symptoms affecting a patient’s emotional state, cognition, and behavior for over two weeks [1]. According to the Diagnostic and Statistical Manual of Mental Disorders, 5th Edition (DSM-5), to be diagnosed with MDD, patients must have a depressed mood or anhedonia that causes social or occupational impairment, along with four or more of the following symptoms: feelings of guilt or worthlessness, lack of energy, poor concentration, appetite changes, psychomotor retardation or agitation, sleep disturbances, or suicidal thoughts. MDD is the most common mood disorder with the Centers for Disease Control and Prevention (CDC) estimating 18.4% of American adults having received this diagnosis during their lifetime [2]. It is considered a public health crisis and a major contributor to morbidity, mortality, disability, and economic stress both in the United States and worldwide [1-4].

The etiology of MDD is multifactorial resulting in central nervous dysregulation from the level of the synapse up to entire neurologic circuits. One long-held theory is the monoamine deficiency hypothesis where a lack of biogenic monoamines at central nerve synapses results in dysfunction and negative symptoms [5-8]. The aptly named selective serotonin reuptake inhibitors (SSRIs) address this etiology by inhibiting the serotonin transporter (SERT) throughout the body. In the central nervous system, this prevents the removal of serotonin from nerve synapses, thereby alleviating both monoamine deficiency and symptoms of MDD [9]. SSRIs are first-line pharmacotherapy for MDD and other psychiatric disorders due to wide tolerance and generally favorable side effect profiles. This class of drugs comprises seven different medications including citalopram, escitalopram, fluoxetine, fluvoxamine, paroxetine, sertraline, and vilazodone [10,11]. Although they share the same mechanism of action, they differ in kinetics, half-life, elimination based on the patient’s age, and drug interactions [12]. In its 2020 Databrief, the CDC estimated 13.2% of American adults had used an SSRI in the past 30 days in 2017-2018, which had increased from 10.6% in 2009-2010. When their data is adjusted for female gender, this increases to 17.7% [13]. In Denmark, there is also increasing SSRI consumption but was estimated lower than in the U.S. at 8.1% of Danish adults in 2021 [14].

Common side effects of SSRIs include gastrointestinal (GI) disturbances, anxiety, agitation, insomnia, sexual dysfunction, and weight gain, with citalopram being the best-tolerated drug followed by fluoxetine, sertraline, paroxetine, and fluvoxamine [10]. Not only that, but SSRIs are well documented to negatively affect platelet hemostasis. Non-specific SERT inhibition prevents serotonin loading into dense granules in platelets. Without serotonin to release upon platelet activation, there is a loss of vasoactive effects that normally facilitate platelet aggregation [15-17]. While SSRI-associated platelet dysfunction is clearly documented to increase risks of GI bleeding, risks are even higher when combined with non-steroidal anti-inflammatory drugs (NSAIDs), agents also known to disrupt platelet aggregation by inhibiting the production of thromboxane A2 [18]. Patients taking such medications have an increased risk of developing upper GI bleeds, although universal guidelines for perioperative usage or specifically for soft-tissue procedures have not been established [19-22]. Auerbach et al. evaluated >500,000 surgical patients at 375 American hospitals and determined an elevated bleeding risk (adjusted odds ratio (aOR) 1.09, 95% CI 1.04-1.15) without including any soft-tissue procedures [23]. Furthermore, holding SSRIs is not without its risks. SSRI discontinuation has well-documented somatic and psychological withdrawal symptoms, most prominently a resurgence of depressive symptoms and worsening anxiety [24-26].

The current literature does not contain a robust review of bleeding complications associated with SSRI consumption for soft-tissue surgical procedures, the primary domain of plastic and reconstructive surgery (PRS). These procedures have the potential for extensive dissection that increases the opportunity for bleeding. Patient commitment and motivation to follow through with post-operative care are also key for successful wound healing, which is key for successful cosmesis. Therefore, it is critical to understand the risk of bleeding when discussing the risks vs benefits of SSRI discontinuation syndrome. In this study, we aim to compile a literature review addressing SSRI usage and post-operative bleeding after PRS and soft-tissue procedures.

## Review

Methods

Search Strategy

A systematic review was performed according to the 2020 Preferred Reporting Items for Systematic Reviews and Meta-Analyses (PRISMA) guidelines [27]. A search was conducted on the National Center for Biotechnology Information (NCBI) PubMed database and Embase. Using the “advanced search” option on PubMed’s website and Embase database, a query was performed to produce the articles with the terms “serotonin uptake inhibitor”/exp AND “plastic surgery”/exp AND “bleeding”/exp. Research published in the English language from 1990 to 2024 was eligible for inclusion. Procedures were limited to those commonly performed by PRS surgeons or those in the same operative field. Only publications with bleeding outcomes were included. Articles in languages other than English, ongoing clinical trials, publications before 1990, literature reviews, and certain observational publications (case reports, background articles, and commentaries) were specifically excluded. The search was run in December 2024.

Data Collection and Analysis

Four different authors (John, Ferri, Brito, and Devineni) reviewed the articles eligible for review. Extracted variables included population demographics, procedural details, and bleeding outcomes. Any discrepancies were resolved after a discussion between the juniors and the senior author (Newman).

Assessment of Risk of Bias

Two different authors (John and Ferri) independently assessed the risk of bias using the Newcastle-Ottawa scale for non-randomized studies [28].

Statistical Analysis

We analyzed the prevalence of SSRI use and the incidence of various bleeding events in the included studies. Due to the limited number of studies and their heterogeneity in terms of the operative field, study design, and outcomes (which precluded the possibility of conducting a meta-analysis), we adopted a qualitative approach to summarize the results instead of abstracting and aggregating data.

Results

Literature Search Results

Using the previously described search strategy, the NCBI query and Embase produced 387 results. These included various articles, including literature reviews, case reports, meta-analyses, retrospective studies, prospective studies, and experimental studies. Two articles were not able to be categorized due to the lack of available abstract or complete text. After removing duplicates and then applying the inclusion and exclusion criteria, five articles were included after the full-text review [29-33]. Basile et al., Harirchian et al., and Harvey et al. were included given that their respective subjects of cosmetic breast and head/neck reconstruction are the domain of PRS [29-31]. Harirchian et al. group together SSRIs and serotonin/norepinephrine receptor inhibitor (SNRI) medication consumption; however, given that both medications limit serotonin reuptake and SNRI usage is generally less frequent, it was not excluded [30]. Harvey et al. evaluate bleeding events by multiple psychiatric medications, including SSRIs, SNRIs, tricyclic antidepressants, benzodiazepines, and a variety of sleep aids. For the focus of this review, only SSRI data from Harvey et al. is presented [31]. Gärtner et al. and Thomsen et al. describe different bleeding outcomes after primary breast oncologic procedures [32,33].

**Figure 1:**
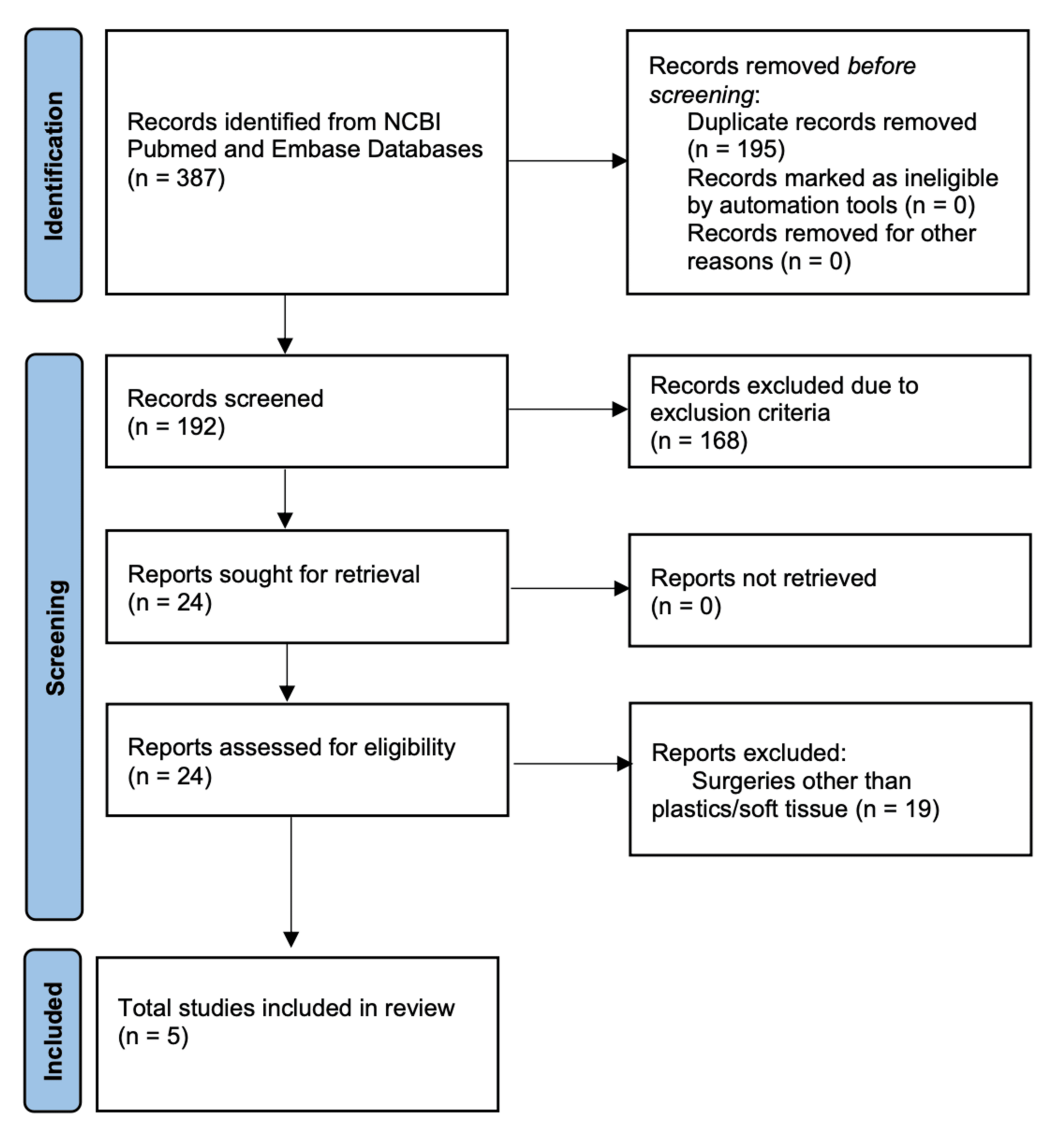
PRISMA study selection process PRISMA: Preferred Reporting Items for Systematic Reviews and Meta-Analyses; NCBI: National Center for Biotechnology Information

Since breast oncologic procedures also involve soft-tissue dissection similar to breast cosmetic procedures, are often part of combination PRS cases, and may be performed by PRS globally, these publications were still included in the analysis. The selection process is summarized in Figure 1. A summary of the findings is described in Tables 1, 2.

**Table 1:** Demographics, procedural details, and bleeding outcomes for SSRI vs non-SSRI consumption for breast procedures *Outcome rare, so odds ratio (OR) approximates risk ratio per study author SSRI: selective serotonin reuptake inhibitor; aRR: adjusted risk ratio; aOR: adjusted odds ratio

Study		Basile et al. (2013) [29]		Gärtner et al. (2010) [32]			Thomsen et al. (2017) [33]	
Design		Retrospective cohort study		Retrospective cohort study			Retrospective cohort study	
Duration		Jan 2001 to Dec 2011		Jan 1996 to Mar 2007			2005 to 2012	
Patient demographics	Total patient number	2,285		14,464			22,238	
	Female	2,285 (100%)		14,464 (100%)			22,238 (100%)	
	Age	17 to >60		<40 to >80			35 to >80	
	SSRI use	Users 196 (8.58%)	Non-users 2,089 (91.42%)	Users 201 (1.38%)	Former users 1,391 (9.61%)	Non-users 12,872 (88.99%)	Users n = 1,080 (4.90%)	Non-users n = 21,158 (95.10%)
Procedure	Augmentation mammoplasty	106	1,056					
	Reduction mammoplasty	28	425					
	Mastopexy	62	608					
	Total mastectomy			656		5,073	384	6,551
	Breast-conserving resection			936		7,799	696	14,607
Outcomes	Total bleeding events	33 (1.44%)		389 (2.60%)			279 (1.30%)	
	Reoperable hematomas	9 (4.59%)	24 (1.15%)	14 (7.0%)	37 (2.6%)	338 (2.70%)		
	Blood product transfusion						19 (1.8%)	260 (1.23%)
Statistical evaluation	p-value	p ≤ 0.001						
	Ratio	OR* 4.14 (95% CI 1.90–9.04)		aRR 2.7 (95% CI 1.6–4.4)	aRR 1.0 (95% CI 0.66-1.3)	aRR 1.0	aOR* 1.2 (95% CI 0.7-1.9	aOR 1.0

**Table 2:** Demographics, procedural details, and bleeding outcomes for SSRI vs non-SSRI consumption for head/neck procedures Procedures included in the studies such as rhytidectomy, subplatysmaplasty, rhinoplasty, submental lipectomy, forehead lift, genioplasty, septoplasty, upper and lower blepharoplasty, and upper eyelid ptosis correction comprise a series of facial esthetic procedures such as nose job, facelift, and neck lifts *Includes both SSRI and SNRI use DDAVP: 1‐deamino‐8‐D‐arginine vasopressin (desmopressin); SSRI: selective serotonin reuptake inhibitor; SNRI: serotonin/norepinephrine receptor inhibitor

Study		Harirchian et al. (2016) [30]		Harvey et al. (2017) [31]	
Design		Retrospective cohort study		Retrospective cohort study	
Duration		Jan 2010 to May 2011		-	
Patient demographics	Total patient number	263		392	
	Female	258 (98.1%)		-	
	Age	61 (44 to 80)		18 to 86	
	SSRI use	Users* 58 (22.1%)	Non-users 205 (77.9%)	Users 54 (13.8%)	Non-users 263 (67.9%)
Procedure	Deep plane rhytidectomy and subplatysmaplasty	181 (68.8%)			
	Revision rhytidectomy	69 (26.2%)			
	Subplatysmaplasty alone	13 (4.90%)			
	Rhinoplasty, rhytidectomy, submental lipectomy, forehead lift, genioplasty, rhinoplasty, septoplasty, upper blepharoplasty, lower blepharoplasty, upper eyelid ptosis correction			392	
Outcomes	Total bleeding events	5 (1.90%)		72 (18.4%)	
	All hematomas	1 (1.72%)	4 (1.95%)		
	"Minor" hematomas	0 (0%)	4 (1.95%)		
	Reoperable hematomas	1 (1.72%)	0 (0%)		
	DDAVP usage			8 (14.8%)	55 (20.9%)
Statistical evaluation	p-value	p = 0.87		p = 0.25	

Assessment of Risk of Bias

To assess the quality of the papers and the risk of bias, the Newcastle-Ottawa scale risk of bias assessment tool was used. The results are compiled in Table 3. Both arms of each study were selected from the cohort of patients seeking soft-tissue intervention, with SSRI consumption retrospectively evaluated. All studies obtained objective categorical data from secure medical records. Each study reports the procedures performed, although only Harvey et al. do not distinguish how many of each was performed. Furthermore, Harvey et al. report that many patients had multiple procedures performed but also do not elaborate further [31].

**Table 3:** Newcastle-Ottawa scale risk of bias assessment Good quality: 3 or 4 stars in the selection domain AND 1 or 2 stars in the comparability domain AND 2 or 3 stars in the outcome/exposure domain. Fair quality: 2 stars in the selection domain AND 1 or 2 stars in the comparability domain AND 2 or 3 stars in the outcome/exposure domain. Poor quality: 0 or 1 star in the selection domain OR 0 stars in the comparability domain OR 0 or 1 star in the outcome/exposure domain SSRI: selective serotonin reuptake inhibitor; SNRI: serotonin/norepinephrine receptor inhibitor

Study	Selection				Comparability	Outcome			Assessment
	Representativeness of the exposed cohort	Selection of the non-exposed cohort	Ascertainment of exposure	Demonstration that outcome of interest was not present at the start of study	Comparability of cohorts on the basis of design of analysis controlled for confounders	Assessment of outcome	Follow-up long enough for outcomes to occur	Adequacy of follow-up of cohorts	
Basile et al. (2013) [29]	Patients who underwent breast cosmetic surgery by a team of two surgeons at a single institution who report SSRI use	Drawn from the same community	Secure records	Yes	Study controls for age, BMI, and additional medications	Secure records	Yes, 60 days	No statement	Good
Harirchian et al. (2016) [30]	All patients who underwent rhytidectomy variations by a single senior surgeon who reported SSRI/SNRI use at a single institution over the reported time frame	Drawn from the same community	Secure records	Yes	Study controls for additional medications and coagulopathies	Secure records	Yes, 8 days	No statement	Good
Harvey et al. (2017) [31]	All patients in 392 consecutive operations by a single senior surgeon who reported psychiatric medication use at a single institution	Drawn from the same community	Secure records	Yes	Study controls for gender	Secure records	Yes	No statement	Poor
Gärtner et al. (2010) [32]	All patients reported in Denmark who had both new diagnosis of breast cancer undergoing index operation and active SSRI use according to mandatory national databases	Drawn from the same community	Secure records	Yes	Study controls for age, additional medications, and 6 comorbidities (liver disease, renal disease, other cancers, thrombocytopenia, autoimmune disease, and vascular disease)	Secure records	Yes, 14 days	Subjects lost to follow-up unlikely to introduce bias	Good
Thomsen et al. (2017) [33]	All patients reported in Denmark who had both new diagnosis of breast cancer undergoing index operation and active SSRI use according to mandatory national databases	Drawn from the same community	Secure records	Yes	Study controls for age, cancer stage, Charlson comorbidity score, and 4 comorbidities (myocardial infarction, congestive heart failure, diabetes, and diabetes with end-organ failure)	Secure records	Yes, 14 days	Subjects lost to follow-up unlikely to introduce bias	Good

Every study controlled for different confounding variables. All studies but Thomsen et al. and Harvey et al. explicitly control for confounding consumption of anticoagulant or antiplatelet medications, with Basile et al. specifically excluding this patient population from their cohort [29,31,33]. While Thomsen et al. do not stratify anticoagulant usage in their cohort, their analysis includes aspirin, NSAID, and statin consumption as other subcategories in their cohort rather than control for them [33]. All the patients in Harvey et al. had a pre-operative coagulation panel and complete blood counts but did not control this data, remark on exclusion, or evaluate for anticoagulant usage [31]. All studies had an appropriate follow-up. Harvey et al. did not require any follow-up because it evaluated specifically intra-operative events [31]. The shortest follow-up otherwise was eight days in Harirchian et al.; given that most post-operative bleeding occurs within the first seven days, this was deemed appropriate [30,34]. No study remarked on the adequacy or percentage of follow-up.

Overall, all studies except for Harvey et al. achieved the highest ranking of “Good.” All studies had an adequately minimal bias in patient selection and evaluation of outcomes [29-33]. Harvey et al. was given a “Poor” ranking because data was only controlled for sex, which is inadequate per the Newcastle-Ottawa guidelines [28,31].

SSRI Prevalence

All studies evaluated SSRI consumption in their cohorts. Active SSRI consumption was determined from hospital medical reconciliations at the time of procedure in Basile et al., Harirchian et al., and Harvey et al. at 8.58%, 22.1%, and 13.1%, respectively [29-31]. Of note, despite being less common medications, Harirchian et al. group both SSRI and SNRI intake together without any subgroup analysis into 22.1% [30]. Differently from SSRIs, SNRIs are both serotonin and norepinephrine reuptake inhibitors, which confers this class of medication with a lower risk of bleeding when compared to SSRIs (34014500). Gärtner et al. and Thomsen et al. are population-based cohorts constructed with Danish national databases. In Denmark, every citizen is assigned a unique civilian personal registration (CPR) number, to which hospitals and pharmacies are required to link the Current Procedural Terminology (CPT) and Anatomical Therapeutic Chemical (ATC) codes associated with all visits, hospitalizations, and prescriptions into national databases. After cross-referencing CPT codes for primary breast cancer index surgeries with SSRI prescriptions from within 30 days, Gärtner et al. and Thomsen et al. estimate SSRI incidents at 1.38% and 4.90% [32,33]. Gärtner et al. additionally evaluate any non-recent history of SSRI at 9.61% by checking any SSRI prescriptions filled greater than 30 days before index surgery [32].

Bleeding Events

The total number of post-operative bleeding events in each cohort was low overall. Basile et al. and Gärtner et al. had bleeding complications (hematoma) in 1.44% and 2.60%, respectively, of their breast cosmetic and oncologic surgical patients [29]. In Harirchian et al., 1.90% of patients had a bleeding complication (hematoma) after facial plastic surgery despite generally “extensive dissection” described by the senior surgeon [30]. Thomsen et al. report transfusion of blood products as its bleeding complication rather than hematoma, but this complication remains rare at 1.30% [33]. Harvey et al. is the only study to focus on intra-operative bleeding rather than post-operative bleeding. 1‐Deamino‐8‐D‐arginine vasopressin (DDAVP) administration acts as the surrogate endpoint for excessive intra-operative bleeding, approximated at 18.4% across the entire cohort [31].

Hematoma Formation and Reoperations

Basile et al. evaluate only reoperable hematomas requiring surgical intervention for breast cosmetic surgery. Nine patients in the SSRI arm (4.59%) required reoperation compared to 24 patients in the non-SSRI arm (1.15%), a significant difference with p < 0.001. There was a 4.14-fold greater bleeding risk due to SSRI consumption (OR 4.14; 95% CI 1.90-9.04). In a subgroup analysis by procedure type, the elevated bleeding risk of SSRI vs non-SSRI persists for breast augmentation (3.7% vs 0.95%), breast reduction (7.14% vs 3.77%), and mastopexy (4.84% vs 1.32%); however, on logistic regression, only augmentation had a statistically significant difference (p < 0.001, 0.3, 0.442) [29]. Gärtner et al. also only evaluate reoperable hematomas requiring surgical intervention for breast oncologic surgery. There were 14 reoperations in the active SSRI arm (7.0%) with 37 in the SSRI history arm (2.6%) and 338 in the non-SSRI arm (2.70%). This results in a 2.7-fold greater bleeding risk due to SSRI consumption (OR 2.7; 95% CI 1.6-4.4) with no change in risk with a history of SSRI (OR 1.0; 95% CI 0.66-1.3) [32].

Harirchian et al. distinguish between “major” hematoma (hematoma requiring reoperation for surgical drainage) and “minor hematoma” (hematoma < 10 cc, which only requires needle aspiration in the office). Both were rare events for facial plastics, with only one major hematoma in the SSRI arm and four minor hematomas in the non-SSRI arm. When comparing the overall hematoma rate of the SSRI and non-SSRI arms, there was no significant difference (1.72% and 1.90%, p = 0.87) [30].

Transfusion Requirements

Thomsen et al. evaluate transfusion requirements after breast oncologic surgery. Two hundred seventy-nine patients required transfusion of at least one unit of packed red cells within 14 days of surgery (1.3%). The transfusion threshold was not reported. The SSRI subgroup had 19 patients requiring transfusion (1.8%) compared to 260 in the non-SSRI subgroup (1.23%). There was no significant difference (OR 1.2; 95% CI 0.7-1.9) [33].

DDAVP Administration

Harvey et al. approximate an elevated risk of intra-operative bleeding by evaluating rates of DDAVP administration (0.3 µg/kg). DDAVP was administered at the discretion of the senior surgeon, who was present during each case and determined that there was a “wet field” based on their three decades of experience. It is this group’s practice to administer DDAVP early based on subjective evaluation of the operative field prior to there being any available objective data (sponge count, labs). Eight of 54 (14.8%) of SSRI users and 55 of 263 non-SSRI users (20.9%) had DDAVP administered without significant differences (p = 0.25) [31].

Discussion

After a literature search, only five publications meeting the criteria were discovered evaluating the link between SSRI consumption and bleeding in PRS and soft-tissue procedures. Five retrospective cohort studies were included with heterogeneous methods and outcomes. Harirchian et al. and Harvey et al. address SSRI-associated bleeding events in facial plastics [30,31]. These studies are comparatively lower powered (~260,400 patients compared to ~2,000-22,000), and each only addresses a single senior surgeon’s patients. The remainder focus on the breast, with Basile et al. focusing on cosmetic surgery and the last two Danish population studies, respectively, addressing bleeding and transfusion requirements in oncologic surgery [29,32,33]. Based on the search conducted both on PubMed and Embase, there have been no studies addressing SSRI-related bleeding in other soft-tissue rearrangement or tissue flap procedures on the torso or extremities.

None of the studies discuss the same confounders or comorbidities, resulting in additional heterogeneity in the analysis. Despite the varying approaches, the post-operative bleeding studies all maintain good standing based on the Newcastle-Ottawa scale risk of bias assessment. Harvey et al. is the outlier in that it minimally controls the data for demographics and does not address any anticoagulant/antiplatelet usage. Furthermore, it does not comment on the prevalence of any comorbidities (chronic kidney disease, von Willebrand disease) for which DDAVP is normally administered. Overall, Harvey et al. is ranked “Poor” with a significant risk of bias [31]. A meta-analysis was unable to be performed on the currently available data due to the heterogeneity in the operative field, design, and outcomes.

SSRI incidence is estimated by each publication. Harirchian et al. has a higher SSRI prevalence than the general population, but given that SNRI medication users were included in the same cohort, it is not possible to determine by how much [30]. Basile et al. and Harvey et al. have the most accurate SSRI prevalence when compared to CDC usage data from the same era [29,31]. This lends credence to the accuracy of Basile et al.’s results as a higher-quality publication. Both Danish population cohorts have lower estimated prevalence compared to Danish usage data, which is unexpected given that a cancer diagnosis would lend to negative mood symptoms. This is confounded by the fact that SSRIs are solely prescription medications in Denmark, so the mandatory national pharmacy database should be accurate. It is, therefore, possible that both Danish studies underestimate SSRI usage and associated bleeding events, but it is unclear by how much [32,33].

All tissue molding-related publications, whether focusing on soft-tissue manipulation in the face or breast, agree that soft-tissue procedures are still associated with a low risk of bleeding complications, ranging from 1.3% to 2.6% [29,32,33]. Basile et al. demonstrate a 4.14-fold increase in bleeding events associated with SSRI consumption, but reoperation was still required <5% [29]. The elevation in bleeding risk after breast surgery was confirmed in the higher-powered Gartner et al. at a 2.7-fold increase, with subgroup analysis demonstrating that this risk comes from use in the past 30 days [32]. Given that Thomsen et al. found no increase in blood product transfusion in a similar cohort, it remains unlikely that the increased bleeding risk is life-threatening [33].

Harirchian et al. did not find any significant difference between bleeding for users and non-users; however, the lower power and rarity of the outcome of interest are limitations of the reliability of its data [30]. Harvey et al. is the only paper that evaluates intra-operative bleeding using DDAVP administration as a surrogate. The methodology of Harvey et al. raises significant doubt about the reliability of their conclusions regarding intra-operative bleeding risk [31]. A Cochrane review of the existing data regarding DDAVP for minimizing intra-operative bleeding suggests low-quality evidence regarding its general intra-operative usage [35]. DDAVP administration, therefore, may not be an appropriate metric for intra-operative bleeding. The previously mentioned lack of evaluation for confounders or effect modifiers severely limits the internal validity of the study. Quantifying intra-operative bleeding based on an evaluation of the “wet field” is inherently subjective and severely limits the external validity of the study. The authors of this study cannot draw any meaningful conclusions from the data as presented [31].

This study, as far as we are aware, is the first systematic review to address the risk of bleeding associated with SSRIs in PRS patients. The limitations of this study lie primarily in only finding five studies addressing this question, making it an early systematic review. Only retrospective cohort studies are available, which only evaluate breast procedures and facial procedures, limiting generalizability. The large cohorts from the Danish studies do strengthen their conclusions. The facial plastic studies are single-center and single-surgeon cohorts, which further limits generalizability. Finally, the significant heterogeneity in the studies precludes a meta-analysis with higher-quality conclusions. Further research should be done on a broader spectrum of procedures, including free flaps, and operative fields to augment the Plastics and Reconstructive Surgeon’s ability to weigh bleeding risks with SSRI use.

## Conclusions

The overall incidence of bleeding complications after soft-tissue procedures appears low. SSRI consumption increases the risk of bleeding complications across a range of cosmetic and oncologic breast procedures. Despite this, the risk of requiring surgical reoperation is <5%, and there is no evidence that these bleeding events are life-threatening. At this juncture, we continue to recommend a thorough discussion between surgeons and patients regarding holding SSRIs as the degree of post-operative complications does not conclusively support risking SSRI discontinuation syndrome. Further randomized studies for holding existing SSRI prescriptions prior to PRS and soft-tissue procedures are needed to definitively investigate this phenomenon. The available data for the effect of SSRI usage on facial plastics is low-powered and questionable, so we are unable to definitively support conclusions in this operative field. Further evaluations of SSRI-related bleeding are recommended for the face/neck, extremities, and other flap procedures.

## References

[1] (2024). 2021 NSDUH Annual National Report. https://www.samhsa.gov/data/report/2021-nsduh-annual-national-report?utm_medium=email&utm_source=transaction.

[2] Lee B, Wang Y, Carlson SA (2023). National, state-level, and county-level prevalence estimates of adults aged ≥18 years self-reporting a lifetime diagnosis of depression - United States, 2020. Morb Mortal Wkly Rep.

[3] Shim RS, Baltrus P, Ye J, Rust G (2011). Prevalence, treatment, and control of depressive symptoms in the United States: results from the National Health and Nutrition Examination Survey (NHANES), 2005-2008. J Am Board Fam Med.

[4] McLaughlin KA (2011). The public health impact of major depression: a call for interdisciplinary prevention efforts. Prev Sci.

[5] Hirschfeld RM (2000). History and evolution of the monoamine hypothesis of depression. J Clin Psychiatry.

[6] Perez-Caballero L, Torres-Sanchez S, Romero-López-Alberca C, González-Saiz F, Mico JA, Berrocoso E (2019). Monoaminergic system and depression. Cell Tissue Res.

[7] Jesulola E, Micalos P, Baguley IJ (2018). Understanding the pathophysiology of depression: from monoamines to the neurogenesis hypothesis model - are we there yet?. Behav Brain Res.

[8] Spellman T, Liston C (2020). Toward circuit mechanisms of pathophysiology in depression. Am J Psychiatry.

[9] Chu A, Wadhwa R (2023). Selective serotonin reuptake inhibitors. StatPearls [Internet].

[10] Ferguson JM (2001). SSRI antidepressant medications: adverse effects and tolerability. Prim Care Companion J Clin Psychiatry.

[11] Edinoff AN, Akuly HA, Hanna TA (2021). Selective serotonin reuptake inhibitors and adverse effects: a narrative review. Neurol Int.

[12] Marken PA, Munro JS (2000). Selecting a selective serotonin reuptake inhibitor: clinically important distinguishing features. Prim Care Companion J Clin Psychiatry.

[13] Brody DJ, Gu Q (2020). Antidepressant use among adults: United States, 2015-2018. NCHS Data Brief.

[14] Ingemann TN, Backe MB, Bonefeld-Jørgensen EC, Skovgaard N, Pedersen ML (2021). Prevalence of patients treated with antidepressant medicine in Greenland and Denmark: a cross-sectional study. Int J Circumpolar Health.

[15] Edinoff AN, Raveendran K, Colon MA (2022). Selective serotonin reuptake inhibitors and associated bleeding risks: a narrative and clinical review. Health Psychol Res.

[16] McCloskey DJ, Postolache TT, Vittone BJ, Nghiem KL, Monsale JL, Wesley RA, Rick ME (2008). Selective serotonin reuptake inhibitors: measurement of effect on platelet function. Transl Res.

[17] Meijer WE, Heerdink ER, Nolen WA, Herings RM, Leufkens HG, Egberts AC (2004). Association of risk of abnormal bleeding with degree of serotonin reuptake inhibition by antidepressants. Arch Intern Med.

[18] Alam SM, Qasswal M, Ahsan MJ, Walters RW, Chandra S (2022). Selective serotonin reuptake inhibitors increase risk of upper gastrointestinal bleeding when used with NSAIDs: a systemic review and meta-analysis. Sci Rep.

[19] Andrade C, Sharma E (2016). Serotonin reuptake inhibitors and risk of abnormal bleeding. Psychiatr Clin North Am.

[20] Jiang HY, Chen HZ, Hu XJ (2015). Use of selective serotonin reuptake inhibitors and risk of upper gastrointestinal bleeding: a systematic review and meta-analysis. Clin Gastroenterol Hepatol.

[21] Anglin R, Yuan Y, Moayyedi P, Tse F, Armstrong D, Leontiadis GI (2014). Risk of upper gastrointestinal bleeding with selective serotonin reuptake inhibitors with or without concurrent nonsteroidal anti-inflammatory use: a systematic review and meta-analysis. Am J Gastroenterol.

[22] Haghbin H, Zakirkhodjaev N, Husain FF, Lee-Smith W, Aziz M (2023). Risk of gastrointestinal bleeding with concurrent use of NSAID and SSRI: a systematic review and network meta-analysis. Dig Dis Sci.

[23] Auerbach AD, Vittinghoff E, Maselli J, Pekow PS, Young JQ, Lindenauer PK (2013). Perioperative use of selective serotonin reuptake inhibitors and risks for adverse outcomes of surgery. JAMA Intern Med.

[24] Zajecka J, Tracy KA, Mitchell S (1997). Discontinuation symptoms after treatment with serotonin reuptake inhibitors: a literature review. J Clin Psychiatry.

[25] Michelson D, Fava M, Amsterdam J, Apter J, Londborg P, Tamura R, Tepner RG (2000). Interruption of selective serotonin reuptake inhibitor treatment. Double-blind, placebo-controlled trial. Br J Psychiatry.

[26] Chiappini S, Vickers-Smith R, Guirguis A, Corkery JM, Martinotti G, Schifano F (2022). A focus on abuse/misuse and withdrawal issues with selective serotonin reuptake inhibitors (SSRIs): analysis of both the European EMA and the US FAERS pharmacovigilance databases. Pharmaceuticals (Basel).

[27] Page MJ, McKenzie JE, Bossuyt PM (2021). The PRISMA 2020 statement: an updated guideline for reporting systematic reviews. BMJ.

[28] (2021). The Newcastle-Ottawa Scale (NOS) for assessing the quality of nonrandomized studies in meta-analyses. https://www.ohri.ca/programs/clinical_epidemiology/oxford.asp.

[29] Basile FV, Basile AR, Basile VV (2013). Use of selective serotonin reuptake inhibitors antidepressants and bleeding risk in breast cosmetic surgery. Aesthetic Plast Surg.

[30] Harirchian S, Zoumalan RA, Rosenberg DB (2012). Antidepressants and bleeding risk after face-lift surgery. Arch Facial Plast Surg.

[31] Harvey D, Punjabi A, Okada H, Zwiebel S, Riazi H, Guyuron B (2018). The incidence of psychiatric medication use and its effect on intraoperative bleeding in facial cosmetic patients. Aesthetic Plast Surg.

[32] Gärtner R, Cronin-Fenton D, Hundborg HH, Pedersen L, Lash TL, Sørensen HT, Kroman N (2010). Use of selective serotonin reuptake inhibitors and risk of re-operation due to post-surgical bleeding in breast cancer patients: a Danish population-based cohort study. BMC Surg.

[33] Thomsen AM, Pedersen AB, Kristensen NR (2017). Use of prescription drugs and risk of postoperative red blood cell transfusion in breast cancer patients: a Danish population-based cohort study. Breast Cancer Res.

[34] Halme AL, Roshanov PS, Tornberg SV, Lavikainen LI, Devereaux PJ, Tikkinen KA (2024). Timing of major postoperative bleeding among patients undergoing surgery. JAMA Netw Open.

[35] Desborough MJ, Oakland K, Brierley C (2017). Desmopressin use for minimising perioperative blood transfusion. Cochrane Database Syst Rev.

